# Entrectinib use in a platinum-refractory mucinous ovarian cancer harboring a NTRK3 gene fusion

**DOI:** 10.1016/j.gore.2023.101187

**Published:** 2023-04-14

**Authors:** Olivia G. Beck, Melissa M. Hardesty

**Affiliations:** a Pacific Northwest University of Health Sciences, 200 University Parkway, Yakima, WA 98901, USA; b Alaska Women's Cancer Care, 3851 Piper Street, Suite U264, Anchorage, AK 99508, USA

**Keywords:** Mucinous ovarian cancer, Entrectinib

## Abstract

Ovarian cancer is difficult to treat, and the mucinous epithelial subtype has a particularly poor response to traditional chemotherapy regimens. Entrectinib is a tumor-agnostic tyrosine kinase inhibitor with limited data regarding its use in ovarian cancers, though it demonstrates significant tumor response and patient tolerability in other settings. Here we outline what we believe to be the first case in which Entrectinib was successfully utilized to treat a patient with mucinous ovarian cancer. A 51-year-old woman with stage IVB mucinous ovarian cancer possessing a KANK1-NTRK3 gene fusion experienced tumor progression and clinical deterioration with conventional chemotherapeutics. Upon initiation of Entrectinib treatment she experienced rapid clinical improvement, with significant partial response and sustained decrease in tumor markers.

## Highlights

• Epithelial ovarian cancer has poor survival outcomes, but targeted chemotherapeutics offer novel options.
• Entrectinib is a tumor agnostic drug with therapeutic benefit in tumors with NTRK gene-fusions.
• Entrectinib successfully upheld durable antitumor response in a mucinous ovarian cancer.

## 1 Introduction

Epithelial ovarian cancer is typically diagnosed at advanced stages with rates of long-term survival less than 20 % (Elias et al., 2018). Stage III/IV mucinous ovarian tumors generally confer adverse prognoses, demonstrating lower responses to standard chemotherapy, with resultant shorter overall survival compared to other histologic subtypes of epithelial ovarian cancer at advanced stage (Bamias et al., 2010). Recently next generation sequencing has allowed identification of individual tumor mutation profiles that reveal potential therapeutic targets. New tumor agnostic regulatory approvals that are focused on the presence of specific genetic therapeutic targets are emerging to offer novel treatment options for some patients (Meagher et al., 2018).

Entrectinib is a tyrosine kinase inhibitor currently FDA approved for metastatic non-small cell lung cancer with ROS1-positivity, as well as for solid tumors with a known neurotrophic tyrosine receptor kinase (NTRK) gene fusion. Entrectinib is also listed as an acceptable therapy for recurrent NTRK gene fusion-positive epithelial ovarian cancers in the NCCN ovarian cancer clinical practice guidelines (Armstrong et al., 2022). It has been shown to impart a durable anti-tumor response, including in cases where disease has previously progressed under first-line chemotherapeutics, with a relatively benign adverse effect profile (Doebele et al., 2019). Fusions of NTRK genes cause oncogenic activation through tyrosine receptor kinases (TRK) associated with downstream signaling pathways like Ras, MAPK, and PI3K. The NTRK gene fusion has a low incidence in ovarian cancer, likely less than 1 %, but nevertheless represents a target for precision therapy (Harbin et al., 2022).

Limited data exist regarding the use of Entrectinib in ovarian cancer. One single case report from 2022 in Japan detailed a case in which Entrectinib was utilized to treat recurrent ovarian cancer with a TPM3-NTRK1 fusion. The drug was unsuccessful, thought to be due to the lack of NTRK protein expression in tumor samples (Endo et al., 2022). Due to Entrectinib’s success in the treatment of other NTRK fusion-harboring cancers, it was used in our case to treat a patient with unresponsive mucinous ovarian cancer with a KANK1-NTRK3 fusion. Here we present the first known published case report of therapeutic efficacy of this agent in ovarian cancer.

## 2 Case report

Written consent was obtained from this patient for the write-up and publication of this case. A 51-year-old native American/Alaska native woman presented to an emergency department with increasing abdominal pain, at which time she was found to have abnormal abdominal imaging. The patient experienced progressive symptoms of abdominal pain, bloating, nausea, vomiting, early satiety, and weight loss during the preceding months.

In the emergency room the patient had a distended abdomen, anemia, leukopenia, low albumin, and elevated alkaline phosphatase. Tumor markers were drawn. A CT scan demonstrated a cystic mass with fine septations in the lower abdomen and pelvis, with largest dimension approximately 20 cm. There was omental caking, carcinomatosis, and ascites, without lymphadenopathy. Imaging also showed hepatic cysts, dilation of intrahepatic biliary ducts, a mass in the pancreatic head with dilation of pancreatic duct, and diffuse bibasilar lung nodules. Due to the presence of a coexisting pancreatic mass and initial uncertainty of primary tumor type, additional imaging was obtained, including a CT abdomen, MRCP, ultrasound, and PET scan. All imaging modalities had similar findings, and multidisciplinary tumor board felt findings were not consistent with pancreatic adenocarcinoma.

Following nondiagnostic biopsies of omentum, pancreas, and lungs, a hysterectomy with bilateral salpingo-oophorectomy and appendectomy was done with sampling of peritoneal deposits. Final pathology revealed the ovarian mass to contain mucinous borderline tumor, with invasive mucinous implants within appendix, fallopian tubes, and peritoneum supportive of a diagnosis of mucinous ovarian primary.

The patient subsequently underwent two cycles of oxaliplatin 130 mg/m^2^ with capecitabine 1000 mg/m^2^ orally on days 1–14, with marked progression of tumor burden. The patient’s pre-treatment CA 19-9 was 2713 U/mL and CA 125 was 235 U/mL. Following two cycles of initial treatment regimen, CA 19-9 rose to 12,937 while CA 125 rose to 303. Chemotherapy cycles were poorly tolerated, and the patient’s health deteriorated, with significant weight loss, intractable nausea and vomiting, and constipation. OmniSeq tumor profiling revealed a KANK1-NTRK3 fusion.

Due to the patient's cachexia, she was admitted to an internal medicine service for nutrition optimization concurrent with initiation of Entrectinib 600 mg PO daily. The patient was TPN dependent and severely malnourished at the time of treatment initiation. Just over four weeks into treatment CA 19-9 levels had fallen to 100, and CA 125 fell to 209. The patient was able to resume tolerance of PO intake and was weaned from TPN. For an additional 12 weeks of treatment, tumor markers continued to decline, with CT scans showing smaller lung lesions with persistent effusions and ascites. After these 12 weeks, Bevacizumab 15 mg/kg was added to target the persistent ascites. With this addition, the frequency at which the patient required paracentesis and thoracentesis decreased from between weekly or every other week, down to between monthly or every other month. The patient reported no adverse effects from Bevacizumab.

Treatment remains ongoing. The patient tolerated Entrectinib therapy well, with the most significant side effect being diarrhea. As of April 2023, the patient has rising tumor markers, though CT has not yet shown progression of disease. Overall, she has experienced 10 months of significant partial response with stable residual disease. The patient has improved from a state of cachectic TPN-dependency back to baseline weight, maintaining appetite and normal eating patterns with satisfactory quality of life.

## 3 Discussion

The incidence of NTRK mutations in ovarian cancers is low yet represents a novel target for chemotherapeutics (Konstantinopoulos et al., 2020). Entrectinib is one such example of targeted NTRK chemotherapy. In original clinical trial data, it was shown to provide a 57.4 % objective response rate, a 50 % partial response rate, a median duration of response of 10.4 months, and a median progression-free survival of 11.2 months. One patient with ovarian cancer with an NTRK gene fusion was included in this trial, without detailed description of outcomes (Doebele et al., 2019). The most common adverse effects of Entrectinib include dysgeusia, dizziness, paresthesia, headache, gastrointestinal symptoms, fatigue, peripheral edema, pyrexia, dyspnea, cough, arthralgias, myalgias, weight gain, and anemia, many of which are dose related. It is a well-tolerated medication with only 4 % of study participants discontinuing its use due to adverse events (Doebele et al., 2019).

Updated data on Entrectinib with longer follow-up and larger sample size found it to provide an objective response rate of 61.2 %, a complete response rate of 15.7 %, and any response in *all* tumors, including two unspecified gynecologic cancers with NTRK mutations. Response rate was found to be higher in patients not previously exposed to systemic chemotherapy. Median progression free survival was found to be 13.8 months and it remained well-tolerated with low rates of cessation (Demetri et al., 2022).

This case demonstrates the promise of Entrectinib in treatment-resistant ovarian cancers harboring an NTRK gene fusion. To our knowledge, only one previous case has reported the use of Entrectinib in any category of ovarian cancer (Endo et al., 2022). Entrectinib was, in that case, ineffective. The lack of response suggests the possibility of a false-positive genetic screening, or that NTRK rearrangements may not always be constitutively expressed (Endo et al., 2022). What we believe to be the first report of successful treatment of mucinous ovarian cancer with an NTRK3 fusion with Entrectinib in our case displays the importance of next generation sequencing, especially in patients experiencing disease progression, intolerability of treatment, or tumor resistance to treatment.

## Author contributions

Olivia G. Beck wrote this manuscript with guidance from Melissa M. Hardesty, MD, MPH. Melissa Hardesty conceived this report, was involved in the direct treatment of the patient, and provided critical feedback and guidance throughout the writing process.

## Declaration of Competing Interest

The authors declare that they have no known competing financial interests or personal relationships that could have appeared to influence the work reported in this paper.
